# Antidiabetic Activity of *Ruellia tuberosa* L., Role of *α*-Amylase Inhibitor: *In Silico*, *In Vitro*, and *In Vivo* Approaches

**DOI:** 10.1155/2015/349261

**Published:** 2015-10-21

**Authors:** Dyah Ratna Wulan, Edi Priyo Utomo, Chanif Mahdi

**Affiliations:** ^1^Academy of Food and Pharmacy Analyst, Putra Indonesia Malang, Street Barito 5, East Java, Malang 65123, Indonesia; ^2^Department of Chemistry, Faculty of Science, Brawijaya University, Street Veteran, East Java, Malang 65145, Indonesia

## Abstract

*Ruellia tuberosa* L. is a folk remedy in the treatment of diabetes mellitus. However, its hypoglycemic activity has not been investigated so far. In the present study, the antidiabetic mechanism of the n-hexane fraction of methanolic extract (HFME) of this plant was investigated *in silico*, *in vitro*, and *in vivo*. *In silico* study was performed using AutoDock4.2 software. *In vitro* *α*-amylase inhibitory activity was investigated by starch-iodine method. A single dose of 450 mg/kg HFME for 14 days was subjected to an antidiabetic screening *in vivo* by a multiple low dose streptozotocin (MLD-STZ) induced rats. Molecular modeling results show that Betulin exhibited noncompetitive *α*-amylase inhibitory activities. The effect of HFME elicited significant reductions of diabetic rat blood glucose. A single dose administration of HFME inhibited *α*-amylase activity *in vivo* (*P* < 0.01) compared to a diabetic control group. Moreover, this extract strongly inhibited the *α*-amylase activity *in vitro* (IC_50_ 0.14 ± 0.005 mg/mL). It is concluded that HFME exerted an antidiabetic effect via *α*-amylase inhibitor. Our findings provide a possible hypoglycemic action of *R. tuberosa* L. as an alternative therapy in the management of diabetes.

## 1. Introduction

Diabetes mellitus (DM), a world large metabolic disorder, is characterized by hyperglycemia. The cause of elevated blood glucose may be associated with genetic, environment component, including insulin resistance and hyperinsulinemia [1]. Patients with diabetes experience significant morbidity and mortality from microvascular and macrovascular complication [2].

Public interest in alternative therapies, including the use of plants and natural dietary supplements, has risen throughout the world.* Ruellia tuberosa* L. is widely disseminated in South East Asia including Indonesia. In folk medicine, it has been used as an antidiabetic, antihypertensive, antipyretic, and analgesic.* R. tuberosa* L. possesses significant blood glucose lowering effect in alloxan-induced diabetic rat and rabbit [3, 4]. It was reported that five flavonoids, cirsimaritin, cirsimarin, cirsiliol 4-glucoside, sorbifolin, and pedalitin along with Betulin, vanillic acid, and indole-3-carboxaldehyde were isolated from the ethyl acetate fraction of methanolic extracts of* R. tuberose* L. [5]. Apigenin, luteolin, 3,5-diglucoside, apigenin-7-O-glucuronide, apigenin glucoside, apigenin rutinoside, luteolin glucoside, and flavone glycoside were also reported in* R. tuberosa* L. [6, 7]. However, its hypoglycemic mechanism of bioactive compounds has not been investigated so far.* In silico* investigation of* R. tuberosa* L. compounds showed that the most potential inhibitor towards rat model *α*-amylase and human *α*-amylase is Betulin. Furthermore, the approximation based on the inhibition constant *k*_*i*_ suggests that Betulin is more potential as an inhibitor *α*-amylase rather than Acarbose [8], but inhibition mechanism of Betulin has not been studied yet. Understanding the mechanism of action and inhibition mechanism of the target enzyme is critical in the early discovery and development of drug candidates. The purpose of a mechanism of action study is to characterize the interaction of a compound with its target, to understand how the compound interacts with the target and how natural substrates at physiological concentrations will modulate this activity.

The aim of this present work is to evaluate the mechanism of antidiabetic effect of an active compound from* R. tuberosa* L.* in silico*,* in vitro*,* and in vivo*. One of the possible diabetic therapy mechanisms is through the inhibition of *α*-amylase. Inhibitors of the carbohydrate digesting enzyme, such as *α*-amylase, now actively searched for the medicine against diabetes, since it could control the postprandial increase of blood glucose [9]. Control of the glucose levels in the blood is the most effective treatment. Long term reduction in hyperglycemia reduces the likehood of developing microvascular and macrovascular complication [10].

## 2. Material and Method

### 2.1. Plant Material and Extract Preparation (HFME)

Aerial parts of* R. tuberosa* L. were collected from their natural habitat in Lawang, East Java, Indonesia. The plant was reidentified by a botanist from the University of Brawijaya, Indonesia. The plant was cleaned and dried under the shade, and the powdered plant (1 Kg) was soaked in methanol (1 L ×3) at room temperature. The methanolic extract was filtered and evaporated using a vacuum rotary evaporator (below 50°C). The crude extract was fractionated with n-hexane (1 : 1). N-Hexane fraction of methanolic extract (HFME) was concentrated using a vacuum rotary evaporator and N_2_. The concentrated HFME was used for an antidiabetic study. The obtained HFME was subjected to a preliminary terpenoid (steroid) screening using thin layer chromatography (TLC) [11]. TLC was performed on silica gel F254, using mobile phase according to Lin et al. [5]. After development, spots were revealed by treatment with Liebermann-Burchard reagent and heating at 110°C for 5 min. Furthermore, Betulin was confirmed with LC-MS [12, 13], and the determination of relative amount (%) of Betulin was based on % peak area.

### 2.2. Animals and Induction of Diabetes

Normal healthy male* Rattus norvegicus* rats (180–220 g) were used for the present investigation. The animal experiments were preceded following the internationally accepted ethical guidelines for the Care of Laboratory Animals. Furthermore, ethical clearance for conducting the studies was obtained from the Animal Care and Use Committee of Brawijaya University (number 232-KEP-UB). The rats were acclimated for one week in our laboratory condition prior to the experiment. Rats were housed under a standard environmental condition at temperature (28 ± 2°C) and a 12 h light/dark cycle. They were fed with standard pellet diet and water ad libitum. Prior to the experiments, they had fasting overnight, but they were still allowed to access water.

For an* in vivo* study, the rats were divided into 3 groups of 6 animal each as follows: normal control (nondiabetic rats administered corn oil 3 mL for 14 days), diabetic control (untreated diabetic rats administered corn oil for 14 days), and therapeutic group (diabetic rats treated with HFME 450 mg/kg b.w. dissolved in 3 mL corn oil for 14 days). Diabetes (diabetic control and therapeutic group) was induced by an intraperitoneal administration of a multiple low dose of streptozotocin (MLD-STZ) at a dose of 20 mg/kg b.w. for five days [16]. Fourteen days after injection, the rats showing Fasting Blood Glucose (FBG) > 250 mg/dL were considered as diabetic and were used for the experiment.

### 2.3. Hyperglycemia Activity

The blood glucose levels of all rats were recorded at regular intervals during the experimental period (0 day and 2nd week). Blood samples were collected from tail vein and blood glucose was measured digitally by glucose meter (GCU meter OneTouch) [17, 18].

### 2.4. *α*-Amylase Inhibitory Assay:* In Vivo* Effect of HFME Administration on Rat Plasma

After an overnight fasting, normal control group, diabetic group, and therapeutic group (15th day after HFME administration) rats were killed by cervical dislocation. Cardiac blood was collected and centrifuged at 5000 ×g for 20 minutes. The rat plasma was collected and stored at 4°C until further use [19, 20]. Amylase activity was carried out according to the Starch-Iodine method. 0.5 mL of substrate buffer solution (0.25 M/L phosphate buffer at pH 7.0 containing 40 mg/dL soluble starch) was mixed well with 100 *μ*L of distilled water and kept at 37°C for 5 minutes. This was done for both the sample and the blank assay. After the incubation period, 0.01 mL of rat plasma was added to the sample solution and well mixed before reincubation at 37°C for 7.5 minutes. After the second incubation, 0.5 mL of coloring reagent (0.254 g iodine and 4.0 g potassium iodide in 1 L) and 2.5 mL of distilled water were added. After mixing, the absorbance of the solution was measured at 581 nm using a spectrophotometer (spectronic 20D). Triplicate assays were performed throughout the study and mean values were used. The unit of amylase activity was arbitrary defined as the following equation:

(1) $$\begin{array}{c} \frac{E_{B1}-E_{S}}{E_{B1}}\times 800, \end{array}$$

where *E*_*S*_ is the absorbance of the starch-iodine complex in sample solution and *E*_*B*1_ is the absorbance of the starch-iodine complex in blank solution.

### 2.5. *α*-Amylase Inhibitory Assay:* In Vitro* Effect of HFME on Rat Plasma

0.10 mL of the serially diluted aqueous plant extract (HFME diluted with DMSO) was incubated with 0.5 mL of substrate buffer solution at 37°C for 5 minutes. The same procedure was adopted as above (using normal control group plasma alpha amylase). The percentage inhibitory activity was calculated using the following formula:

(2) $$\begin{array}{c} \frac{A-B}{B}\times 100 \end{array}$$

*A* is the activity of the enzyme with test solution and *B* is the activity of the enzyme without test solution. The percentage inhibitory activity of alpha amylase then converted to IC50. To compare* in vitro* and* in silico* result, IC50 converted to *k*_*i*_ by calculation reported in Cer et al. [21].

### 2.6. *In Silico* Inhibitory Mechanism

Three main mechanisms of inhibition are competitive, noncompetitive, and uncompetitive. The inhibitory mechanism involves protein and multiple ligand (substrate, inhibitor, and cofactor) interaction. Protein-multiple ligand interaction could be established using multiple ligand simultaneous docking (MLSD) strategies. One of the strategies is Lamarkian genetic algorithm (LGA) using AutoDock4 [22].

Docking was started with the random initialization of population. Different from the single-ligand docking, an individual solution in the multiligand docking contains conformations of multiple ligands. Multiligand docking program loads in PDBQT input file for each ligand (inhibitor and substrate). Each ligand object will be randomly initialized with its own set of state variables. In this manner, each ligand object has its own configuration, ligand center, and torsion tree. Standard LGA procedure is applied. The total interaction energy and inhibition constant between all ligands and protein receptor are computed using modified energy function of AutoDock4.2.

Betulin, an *α*-amylase inhibitor in* R. tuberose*, as a ligand [8], and maltose as a substrate were docked into* Rattus* *α*-amylase model. The* Rattus* *α*-amylase model is created according to Ratna Wulan et al. [8]. The study about inhibition mechanism (competitive, uncompetitive, or noncompetitive) of Betulin has not been published yet. Here, we suggest the inhibition mechanism of Betulin using* in silico* approach. In noncompetitive inhibition, *k*_*i*_ of enzyme-substrate complex, enzyme-inhibitor complex, and enzyme-substrate-inhibitor complex undergos (4). In competitive or uncompetitive inhibition, *k*_*i*_ of enzyme-substrate complex, enzyme-inhibitor complex, and enzyme-substrate-inhibitor complex failed to complete (4). To validate noncompetitive inhibition approach that was used, we calculate *k*_*i*_ of enzyme-substrate complex, enzyme-inhibitor complex, and enzyme-substrate-inhibitor complex using (4) from several small *α*-amylase inhibitors such as Betulinic acid, Curcumin, and Bisdemethoxycurcumin [14, 15, 23].

#### 2.6.1. Enzyme Substrate Inhibitor Reaction and Equation

**Scheme 1:**
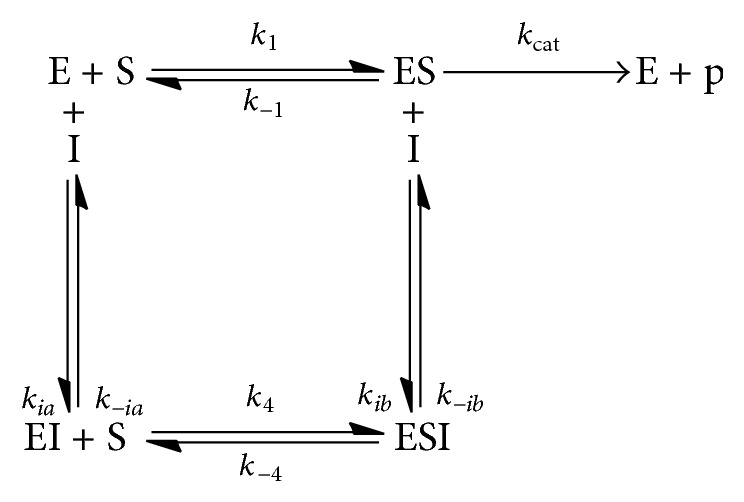


*For Noncompetitive Inhibition.* Inhibitor (I) binds equally well to both free enzyme (E) and the enzyme-substrate complex (ES) (see Scheme 1). These binding events occur exclusively at a site distinct from the precise active site occupied by substrate, where *K*_*ia*_ = *k*_−*ia*_/*k*_*ia*_ and *K*_*ib*_ = *k*_−*ib*_/*k*_*ib*_. Note that we are assuming that the formation of both enzyme complexes is in equilibrium with the respective substrate/inhibitor and that *K*_1*a*_, *K*_1_, *K*_4_, and *K*_1*b*_ are* association* constants. Association constants are reported as inhibition constants calculated by AutoDock4.2. The reaction equations are then as follows:

(3) Kb=ESIESI;K1=ESES;K4=ESIEIS;Ka=EIEI.

Then, substitution of [ESI], [ES], [EI], [E], and [I] make the following final equation:

(4) $$\begin{array}{c} K_{b}=\frac{K_{4}K_{a}}{K_{1}}. \end{array}$$

Noncompetitive inhibitor mechanism should pose (4) above.

**Scheme 2:**
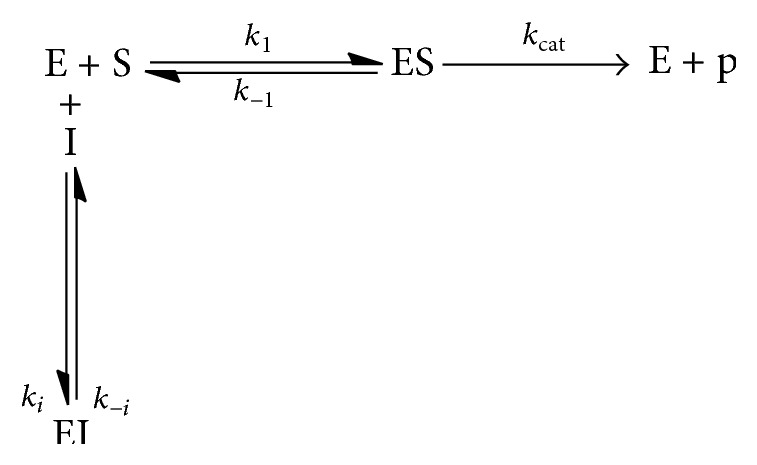


*For Competitive Inhibition.* The inhibitor binds to the active site and prevents binding of the substrate. A competitive inhibitor binds only to the free enzyme (see Scheme 2).

**Scheme 3:**
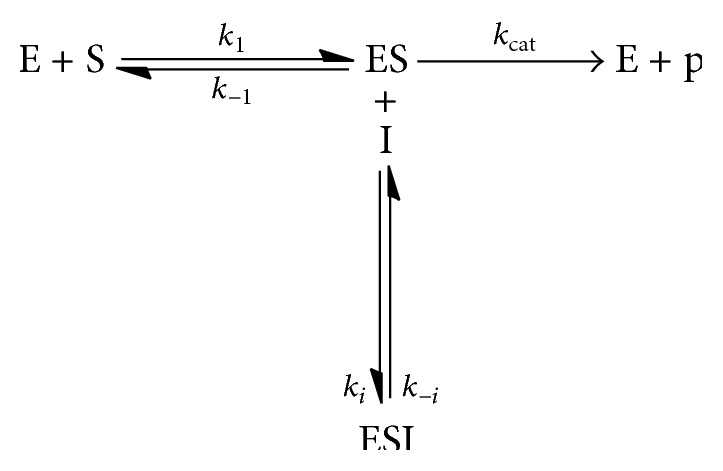


*For Uncompetitive Inhibition.* In the case of uncompetitive inhibition, the inhibitor binds to the E-S complex and prevents conversion to the product (see Scheme 3).

### 2.7. Statistical Analysis

The results of* in vivo* inhibitory activity of *α*-amylase were expressed as mean ± SEM and were analyzed using one-way analysis of variance (ANOVA) followed by least significant difference (LSD). ANOVA and LSD were conducted using SPSS version 15.0. Differences were considered significant at *P* < 0.01. The IC50 values were determined from plots of percent inhibition versus concentration and were calculated by probit regression analysis of the mean inhibitory values. The IC50 values were defined as the concentration of the extract, containing the *α*-amylase inhibitor that inhibited 50% of the rat pancreatic *α*-amylase activity.

## 3. Results

### 3.1. Preparation of the HFME and Betulin Characterization

The methanol extract of dried aerial part of* R. tuberosa* L. (1 Kg) was portioned with n-hexane to obtain HFME. The percentage yield of HFME was 1.69% relative to dried aerial part. The preliminary steroid screening revealed the presence of steroid. The presence of steroids was detected visually as violet spots after spray with Liebermann-Burchard reagent. Betulin (steroid) in HFME was further identified by comparing their spectroscopic data with literature value. The mass spectral analysis of the liquid chromatography of HFME sample revealed the presence of Betulin. The molecular ion peak of Betulin that was found at* m/z* 441 [M-H]^−^ was noticed. The percentage (W/W) amount of Betulin was found to be 6.96% in HFME.

### 3.2. Effect of HFME on *α*-Amylase Activity* In Vitro*

**Figure 1:**
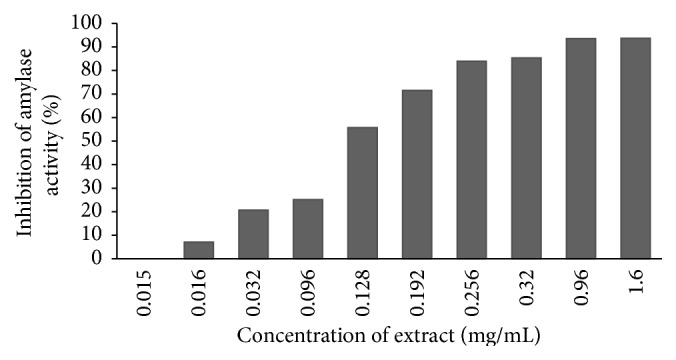
Amylase inhibitory activity of HFME* in vitro*.

Data obtained on the *α*-amylase inhibition effect of HFME is shown in Figure 1. HFME was found to inhibit *α*-amylase activity in rat plasma from 7.13% to 93.77% with increasing graded concentration of the extract (0.016 to 1.6 mg/mL). In addition, the inhibitory effect of HFME in rat *α*-amylase serum appeared to be concentration dependent following sigmoid curve, although there was no significant (*P* > 0.05) effect in lower concentration (0.015 mg/mL). The IC50 was 140 ± 53 *μ*g/mL as shown in Table 1. Based on noncompetitive inhibition equation reported in Cer et al. [21], the conversion IC50 values to *k*_*i*_ is 314 *μ*M.

### 3.3. Effect of HFME Administration on *α*-Amylase Activity* In Vivo*

*In vitro* enzyme-inhibitor effect is not always reproducible* in vivo*. It was thus necessary to confirm effectivity of the observed results* in vivo*. This was done by investigating Fasting Blood Glucose (FBG) in normal control, diabetic control, and therapeutic rat groups and investigating *α*-amylase unit activity. Diabetes was confirmed when hyperglycemia was detected.

The effect of HFME on amylase unit activity is shown in Table 2. Oral administration of HFME were found to possess significant (*P* < 0.01) *α*-amylase inhibitory activity in therapeutic group. It seems that EF containing Betulin can inhibit *α*-amylase* in vivo*.

The effect of oral administration of HFME of* R. tuberosa* L. is shown in Table 3. There was a significant elevation of blood glucose in diabetic control group when compared with normal control group. Administration of HFME of* R. tuberosa* L. 450 mg/kg b.w. for two week tends to bring the parameters significantly towards the normal control group. Our study suggests that the reported hypoglycemic effect of HFME of* R. tuberosa* L. may, in part, be mediated through the inhibition of *α*-amylase activity.

### 3.4. *In Silico* Inhibitory Mechanism

**Figure 2:**
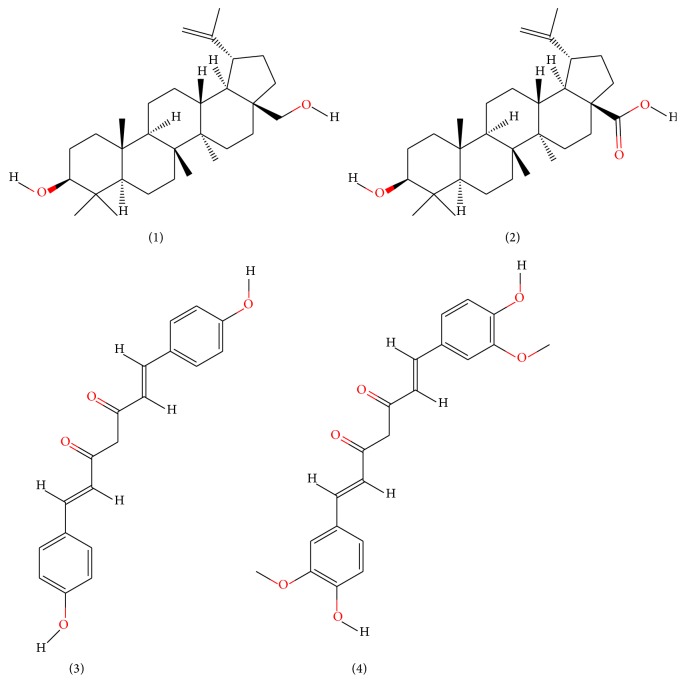
Chemical structure of (1) Betulin, MW 442.72 g/mol; (2) Betulinic acid, MW 456.70 g/mol; (3) Bisdemethoxycurcumin, MW 308.32 g/mol; (4) Curcumin, MW 368.37 g/mol.

Betulin, Betulinic acid, Bisdemethoxycurcumin, and Curcumin (Figure 2) had binding ability towards *α*-amylase.* In silico* study results (Table 4) showed that Betulin has higher binding affinity to *α*-amylase (*k*_*i*_ Betulin 13.12 *μ*M; *E*_binding_  −6.66 kcal/mol) than Betulinic acid (*k*_*i*_ 75.66 *μ*M; *E*_binding_  −5.62 kcal/mol), Bisdemethoxycurcumin (*k*_*i*_ 45.86 *μ*M; *E*_binding_  −5.92 kcal/mol), and Curcumin (*k*_*i*_ 260.62 *μ*M; *E*_binding_  −4.89 kcal/mol). It also shows that Betulinic acid and Betulin undergone (4), while Bisdemethoxycurcumin and Curcumin failed to follow (4). Furthermore, the amino acid residues interacting with the binding site of *α*-amylase were hydrogen bonds.

## 4. Discussion

The results of the present study reveal that the treatment HFME of* R. tuberosa* L. shows potent inhibition of *α*-amylase activity* in vitro*.* Ruellia tuberosa* L. is gaining much importance in diabetic control, since phytochemical analysis has shown the presence of steroid (Betulin) in HFME. Karthic et al. [14] has also reported that the compound of aqueous extract* S. cumini*, Betulinic acid, has a structure similarity with Betulin showing inhibitory activity to *α*-amylase.

Studies regarding interaction between Inhibitor and *α*-amylase have significance in obtaining a better insight in the mechanism of *α*-amylase inhibition. Thus, the molecular docking predictions were used to find the probable inhibition mechanism. Ratna Wulan et al. [8] have also reported* in silico* study that Betulin, steroid compound of* R. tuberosa* L., is an *α*-amylase inhibitor. Bisdemethoxycurcumin, Curcumin, Betulinic acid, and Betulin have a small *E*_bingding_, and a small inhibition Constanta (*k*_*i*_). Small values of *k*_*i*_ and *E*_bingding_ verify that the ligand is efficient with more affinity towards *α*-amylase, suggesting that the ligands are *α*-amylase inhibitor. The results are in agreement with the previous studies on the* in vitro* study [14, 15, 23]. Bisdemethoxycurcumin (uncompetitive inhibitor) and Curcumin (competitive inhibitor) failed to complete (4). Betulinic acid (noncompetitive inhibitor) undergoes (4). These conditions show that (4) can be used to predict noncompetitive *α*-amylase inhibitor mechanism. Furthermore, Betulin undergoes (4), suggesting its noncompetitive mechanism. The docking results revealed that no Van der Waals, hydrophobic, or electrostatic interactions existed, but only hydrogen bond played a major role in the binding of Betulin, Bisdemethoxycurcumin, and Curcumin against *α*-amylase. It is necessary to convert IC50 into *k*_*i*_ for comparing* in silico* and* in vitro* study results. Based on the calculation using noncompetitive inhibition, *k*_*i*_ value of Betulin from* in vitro* study was 314 *μ*M, which is nearly 24 times bigger than *k*_*i*_ result* in silico*.

Calculated *k*_*i*_ value from* in vitro* study depends on the concentrations of the enzyme (or target molecule), the inhibitor, and the substrate along with other experimental conditions.* In silico* *k*_*i*_ value, an intrinsic thermodynamic quantity, is independent of the substrate but depends on the enzyme (target) and inhibitor. These matters make a difference in the value of *k*_*i*_ *in vitro* and* in silico*.

For confirmation of the* in vitro* and* in silico* results, we have performed* in vivo* study using diabetic rat induced by streptozotocin (STZ). STZ induces chemical diabetes in experimental animal [14]. STZ liberates, and participates in DNA damage; as a result pancreatic *β*-cells undergo the destruction by necrosis [24, 25]. Damage of pancreatic *β*-cells results in a decrease in endogenous insulin release, which paves the way for the decreased utilization of glucose by the tissues. In this study, induction diabetes of STZ causes a significant elevation in the level of blood glucose in rats. Some studies report that MLD-STZ causes diabetes type 1 [26]. Type 1 diabetes is due to an autoimmune destruction of the insulin-producing pancreatic *β*-cells leading to the lack of insulin production. One of strategies method adopted to cure diabetes mellitus involves the inhibition of carbohydrate digesting enzyme such as *α*-amylase, thereby lowering postprandial glucose level [9]. In this study, the effect of HFME on the activities of *α*-amylase* in vivo* was evaluated. Administration of 450 mg/kg b.w. of HFME of* Ruellia tuberosa* L. significantly decreases the FBG after two weeks of treatment in these rats, suggesting that it has hypoglycemic properties. This result is in agreement with the previous studies on the* in vivo* antidiabetic potential of* R. tuberosa* L. mentioning that the extracts of this plant possessed strong glucose lowering property in alloxan-induced diabetic rat and rabbit [3, 4]. Alloxan and STZ induce diabetes in different mechanisms, both causing a significant elevation of blood glucose level and inducing type 1 diabetes [27]. HFME containing Betulin is highly lipophilic and may easily cross membrane and exert its pharmacological effects such as inhibiting *α*-amylase. Inhibitors of *α*-amylase delay the breaking down of carbohydrate and diminish the postprandial blood glucose excursion in a person suffering from diabetes. Furthermore in this study, oral administrations of HFME were found to possess significant (*P* < 0.01) *α*-amylase inhibition activity in therapeutic group compared with normal control and diabetic control groups. The ability of inhibiting *α*-amylase* in vivo* by the HFME indicates control of FBG mediated through the inhibition of carbohydrate digestion.

## 5. Conclusion

In conclusion, the results of this study indicate that one of the mechanisms by which* R. tuberosa* L. exhibited its hypoglycemic potential is through the inhibition of pancreatic *α*-amylase. According to the* in silico* studies, it acts as a noncompetitive inhibitor of the *α*-amylase. However, further study is needed to isolate the Betulin and to verify noncompetitive inhibitor* in vitro*. Last but not least, this study provides scientific support to use* R. tuberosa* L. for the treatment of DM.

## Figures and Tables

**Table 1:** IC50 of HFME.

P_*n*_	P_1_	P_2_	P_3_	P_4_	P_5_	P_6_
IC50 (mg/mL)	0,15	0,24	0,11	0,08	0,14	0,14
Average IC50 (mg/mL)	0,14 ± 0,055					

The values are expressed as means ± SEM of six tests. P_*n*_ = plasma amylase of rat control group.

**Table 2:** Effect of HFME on amylase unit activity *in vivo.*

Groups	Control	Diabetic control	Therapeutic
Amylase unit activity	404,6 ± 5,37	386,6 ± 31,25	228,3 ± 68,70^*∗*^

Values are given as mean ± SD for groups of six animals each. Values are statistically significant ^*∗*^ *P* < 0.01.

**Table 3:** Effect of HFME on Fasting Blood Glucose (FBG) of control, diabetic, and therapeutic rats.

Rat groups	Blood glucose (mg/dL)	
	0 day	2nd week
Control	111 ± 7,9	113 ± 10,5
Diabetic control	332 ± 118,8^*∗*^	314 ± 98,9^*∗*^
Therapeutic	399 ± 82,7^*∗*^	114 ± 21,3

Values are given as mean ± SD for groups of six animals each. Values are statistically significant ^*∗*^ *P* < 0.01.

**Table 4:** *In silico* study results.

Ligand name/PubChem ID	Substrate	Target molecule (PDB ID)	*k*_*i*_ EI complex (*µ*M)	*E*_binding_ of EI complex (kcal/mol)	Docking score (3)	Predicted inhibition mode	Inhibition mode in the previous study	Reference	H-bond interaction	Graphical image of ligand-molecule complex
Betulin/CID 72326	Maltose	*Rattus* alpha amylase model	13.66	−6.66	23.36 *µ*M $=\frac{938020\;µ\text{M}\times 13.66\;µ\text{M}}{427180\;µ\text{M}}$ 23.36 *µ*M ≈ 29.99 *µ*M	Noncompetitive	—	—	ASN374, ASN374, ASN376	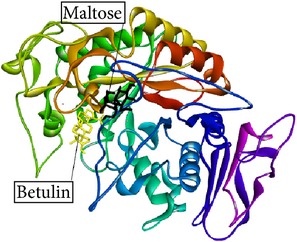

Betulinic acid/CID 64971	Maltose	Porcine pancreatic *α*-amylase (1.ose)	75.66	−5.62	149.13 µM $\approx \frac{942460\;µ\text{M}\times 75.66\;µ\text{M}}{493240\;µ\text{M}}$ 149.13 *µ*M ≈ 144.26 *µ*M	Noncompetitive	Non competitive	Karthic et al. [14]	—	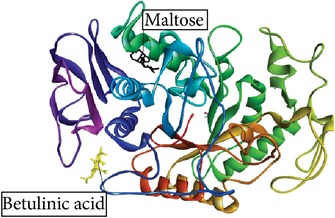

Bisdemethoxycurcumin/ CID 5315472	Maltose	Human alpha amylase (3.old)	45.86	−5.92	83.49 µM $\neq \frac{682600\;µ\text{M}\times \;45.86\;µ\text{M}}{791530\;µ\text{M}}$ 83.49 *µ*M ≠ 39.55 *µ*M	Un competitive or competitive	Un competitive	Najafian [15]	ARG389	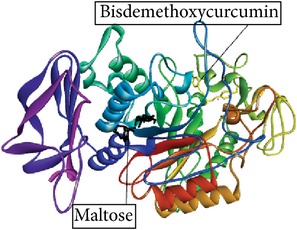

Curcumin/CID 969516	Maltose	Human alpha amylase (3.old)	260.62	−4.89	841.04 µM $\neq \frac{132.63\;µ\text{M}\times \;260.62\;µ\text{M}}{791530\;µ\text{M}}$ 841.04 *µ*M ≠ 0.044 *µ*M	Un competitive or competitive	Competitive	Karthic et al. [14]	LYS 200, GLY 306, HIS 201	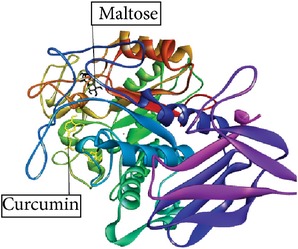

Graphical image showed interaction between ligand-substrate and enzyme complexes. Yellow color is ligand, black color is substrate, and whole structure is of amylase.

## References

[1] Ezenwaka C. E., Kalloo R., Uhlig M., Eckel J. (2004). Relationship between adiponectin and metabolic variables in Caribbean offspring of patients with type 2 diabetes mellitus. *Hormone and Metabolic Research*.

[2] DeFronzo R. A. (1999). Pharmacologic therapy for type 2 diabetes mellitus. *Annals of Internal Medicine*.

[3] Rajan M., Kishor Kumar V. K., Satheesh Kumar P. S., Swathi K. R., Haritha S. (2012). Antidiabetic, antihyperlipidaemic and hepatoprotective activity of methanolic extract of *Ruellia tuberosa* Linn leaves in normal and alloxan induced diabetic rats. *Journal of Chemical and Pharmaceutical Research*.

[4] Shahwara D., Ullah S., Ahmad M., Ullah S., Ahmad N., Khan M. A. (2011). Hypoglycemic activity of *Ruellia tuberosa* linn (Acanthaceae) in normal and alloxan-induced diabetic rabbits. *Iranian Journal of Pharmaceutical Sciences*.

[5] Lin C. F., Huang Y. L., Cheng L. Y. (2006). Bioactive flavonoids from *Ruellia tuberosa*. *The Journal of Chinese Medicine*.

[6] Nair A. G. R., Subramanian S. S. (1974). Apigenin glycosides from *Thunbergia fragrans* and *Ruellia tuberosa*. *Current Science*.

[7] Wagner H., Danninger H., Iyengar M. A. (1971). Synthesis of glucuronides in the flavonoid-series. 3. Isolation of apigenin-7-D-glucuronide from *Ruellia tuberosa* L. and its synthesis. *Chemische Berichte*.

[8] Ratna Wulan D., Priyo Utomo E., Mahdi C. (2014). Molecular modeling of * Ruellia tuberosa* L compounds as a-amylase inhibitor: an* in silico* comparation between human and rat enzyme model. *Bioinformation*.

[9] Tundis R., Loizzo M. R., Menichini F. (2010). Natural products as *α*-amylase and *α*-glucosidase inhibitors and their hypoglycaemic potential in the treatment of diabetes: an update. *Mini-Reviews in Medicinal Chemistry*.

[10] Attele A. S., Zhou Y.-P., Xie J.-T. (2002). Antidiabetic effects of *Panax ginseng* berry extract and the identification of an effective component. *Diabetes*.

[11] Harborne J. B. (1998). *Phytochemical Methods: A Guide to Modern Techniques of Plant Analysis*.

[12] Rhourri-Frih B., Chaimbault P., Claude B., Lamy C., André P., Lafosse M. (2009). Analysis of pentacyclic triterpenes by LC–MS. A comparative study between APCI and APPI. *Journal of Mass Spectrometry*.

[13] Hu Z., Guo N., Wang Z. (2013). Development and validation of an LC-ESI/MS/MS method with precolumn derivatization for the determination of Betulin in rat plasma. *Journal of Chromatography B: Analytical Technologies in the Biomedical and Life Sciences*.

[14] Karthic K., Kirthiram K. S., Sadasivam S., Thayumanavan B., Palvannan T. (2008). Identification of *α*-amylase inhibitors from *Syzygium cumini Linn* seeds. *Indian Journal of Experimental Biology*.

[15] Najafian M. (2015). The effects of curcumin on alpha amylase in diabetics rats. *Zahedan Journal of Research in Medical Sciences*.

[16] Lukiati B., Aulanni'am, Darmanto W. (2012). The effects of *Curcuma heyneana* ethanolic extract on the superoxide dismutase activity and histological pancreas of type 1 diabetes mellitus rats. *International Journal of Basic & Applied Sciences*.

[17] Rajan M., Kumar V. K., Kumar P. S., Ramaniyam R. T., Kumar N. S. (2010). Antidiabetic activity of ethanolic extract on *Albizia odoratissima* (If) benth in alloxan induced diabetic rats. *International Journal of Pharmaceutical Science*.

[18] Friedewald W. T., Levy R. I., Fredrickson D. S. (1972). Estimation of the concentration of low-density lipoprotein cholesterol in plasma, without use of the preparative ultracentrifuge. *Clinical Chemistry*.

[19] Kobayashi K., Baba E., Fushiya S. (2003). Screening of mongolian plants for influence on amylase activity in mouse plasma and gastrointestinal tube. *Biological and Pharmaceutical Bulletin*.

[20] Kobayashi K., Funayama N., Suzuki R., Yoshizaki F. (2002). Survey of the influence of Chinese medicinal prescriptions on amylase activity in mouse plasma and gastrointestinal tube. *Biological and Pharmaceutical Bulletin*.

[21] Cer R. Z., Mudunuri U., Stephens R., Lebeda F. J. (2009). IC50-to-Ki: A web-based tool for converting IC50 to Ki values for inhibitors of enzyme activity and ligand binding. *Nucleic Acids Research*.

[22] Li H., Li C. (2010). Multiple ligand simultaneous docking: orchestrated dancing of ligands in binding sites of protein. *Journal of Computational Chemistry*.

[23] Ponnusamy S., Zinjarde S., Bhargava S., Rajamohanan P. R., Ravikumar A. (2012). Discovering Bisdemethoxycurcumin from *Curcuma longa rhizome* as a potent small molecule inhibitor of human pancreatic *α*-amylase, a target for type-2 diabetes. *Food Chemistry*.

[24] Rerup C. C. (1970). Drugs producing diabetes through damage of the insulin secreting cells. *Pharmacological Reviews*.

[25] Eleazu C. O., Eleazu K. C., Chukwuma S., Essien U. N. (2013). Review of the mechanism of cell death resulting from streptozotocin challenge in experimental animals, its practical use and potential risk to humans. *Journal of Diabetes & Metabolic Disorders*.

[26] Szkudelski T. (2001). The mechanism of alloxan and streptozotocin action in B cells of the rat pancreas. *Physiological Research*.

[27] Lukić M. L., Stošić-Grujičić S., Shahin A. (1998). Effector mechanisms in low-dose streptozotocin-induced diabetes. *Developmental Immunology*.

